# Anterior reconstruction using femoral ring allografts for thoracolumbar infectious spondylitis: Clinical and radiologic outcomes of a retrospective cohort study

**DOI:** 10.1097/MD.0000000000051153

**Published:** 2026-11-06

**Authors:** Myung-Jin Sung, Chun-Gon Park, Hyoung-Yeon Seo

**Affiliations:** a Department of Orthopedic Surgery, Chonnam National University Hospital, Gwangju, Republic of Korea; b Department of Orthopedic Surgery, Chonnam National University Medical School, Gwangju, Republic of Korea.

**Keywords:** anterior reconstruction, corpectomy, femoral ring allograft, infectious spondylitis, thoracolumbar spine

## Abstract

Surgical treatment is required in infectious spondylitis when conservative management fails or vertebral destruction causes instability. Autologous bone graft is considered the gold standard but is limited by donor-site morbidity and availability. Femoral ring allografts are widely used in degenerative conditions, but their role in infectious spondylitis is unclear. This study evaluated the clinical, radiologic, and laboratory outcomes of anterior reconstruction using femoral ring allografts. In this single-center retrospective cohort study, reported in accordance with the Strengthening the Reporting of Observational Studies in Epidemiology statement, we reviewed 25 patients who underwent anterior corpectomy and reconstruction using femoral ring allografts followed by posterior instrumentation between December 2011 and September 2023. Clinical outcomes (VAS, Oswestry Disability Index, Frankel grade), laboratory parameters (erythrocyte sedimentation rate, C-reactive protein), and radiologic outcomes (Cobb angle, fusion) were compared preoperatively and at final follow-up. Paired comparisons used the Wilcoxon signed-rank test, with Hodges–Lehmann estimates, distribution-free 95% confidence intervals (CIs), and effect sizes (*r*). Infection control and complications were analyzed. The mean follow-up was 29.2 ± 23.9 months (range, 12.0–87.2 months). VAS improved from 7.3 ± 1.1 to 1.9 ± 1.0 (Hodges–Lehmann estimate −5.50; 95% CI, −6.00 to −5.00; *r*  = 0.87) and Oswestry Disability Index from 37.7 ± 5.9 to 10.7 ± 3.5 (−26.50; 95% CI, −29.50 to −23.50; *r* = 0.87; both *P* < .001). Neurological function improved significantly (*r* = 0.76, *P* < .001). Erythrocyte sedimentation rate and C-reactive protein decreased significantly (both *P* < .001). Sagittal alignment was corrected with partial loss over time but remained improved at final follow-up (5.55°; 95% CI, 3.40°–8.95°; *r* = 0.69; *P* < .001). Fusion was achieved in 92% and infection control in 96%. Complications occurred in 16%. In patients with large anterior column defects not amenable to tricortical iliac autograft, femoral ring allograft reconstruction achieved reliable infection control, a high fusion rate, and satisfactory clinical and radiologic outcomes over a mean follow-up of 29.2 months. Within this period, it appears a reasonable alternative to structural autograft when donor-site morbidity or graft availability is a concern. Longer-term comparative studies are needed to confirm the durability of graft incorporation and to define its role relative to contemporary titanium-based reconstruction.

## 1. Introduction

Spinal infection, including vertebral osteomyelitis and infectious spondylodiscitis, remains a clinically challenging condition associated with substantial morbidity. Hematogenous spread is the predominant pathophysiologic mechanism; however, the incidence of spinal infections has increased steadily in recent years owing to population aging, a higher prevalence of immunocompromised states, and the growing use of spinal interventions and invasive procedures.^[1-4]^ Contemporary epidemiologic data from Europe have demonstrated a marked rise in vertebral osteomyelitis over the past decade.^[3,4]^

Back pain is the most common presenting symptom, frequently accompanied by elevated inflammatory markers.^[1,2]^ If inadequately treated, progressive vertebral destruction may result in mechanical instability, deformity, and neurologic compromise. Although most patients can be managed conservatively with targeted antimicrobial therapy and immobilization,^[5,6]^ surgical treatment is mandatory in patients presenting with intractable pain, progressive neurologic compromise, epidural abscess formation, structural instability, significant vertebral body destruction, or failure of conservative treatment.^[5,7]^

When bony destruction is limited, debridement with or without limited stabilization may suffice.^[6]^ However, in advanced infectious spondylitis characterized by extensive vertebral body collapse, anterior column reconstruction combined with posterior instrumentation is frequently required to restore structural integrity and sagittal alignment.^[7,8]^ In such cases, the selection of reconstructive material remains a matter of debate.

Autologous tricortical iliac or fibular bone grafts have traditionally been regarded as the gold standard due to their osteogenic potential and resistance to infection.^[8]^ However, harvesting large structural autografts is associated with donor-site morbidity, including persistent pain, hematoma, neurovascular injury, and limited graft availability, particularly when long-segment reconstruction is required.^[9]^

Allograft reconstruction has emerged as a viable alternative that provides adequate structural support while avoiding donor-site morbidity. Comparative studies evaluating autografts and allografts in spinal reconstruction have shown mixed results. Some authors have reported superior fusion with autografts, whereas others demonstrated no significant difference in clinical or radiologic outcomes between autograft and allograft use.^[10,11]^ Structural allografts, including tibial and fibular strut grafts, have demonstrated acceptable fusion and infection control rates in infectious spondylitis.^[12-14]^ More recently, titanium-based reconstruction has become the more frequently reported option in this setting, and recent meta-analyses of spinal tuberculosis have found no significant difference in fusion, deformity correction, or complications between titanium mesh grafting and structural bone grafting.^[15,16]^

Femoral ring allografts have been widely utilized in anterior lumbar interbody fusion and revision spine surgery because of their robust cortical structure and favorable load-bearing characteristics.^[17]^ Their circular geometry provides substantial axial strength and resistance to collapse, making them particularly attractive for large anterior column defects. However, despite their biomechanical advantages, clinical data regarding the use of femoral ring allografts in infectious spondylitis remain limited.

Given the paucity of literature specifically addressing femoral ring allograft reconstruction in advanced infectious spondylitis, further clinical evidence is required. The purpose of this study was to evaluate the clinical, radiologic, and laboratory outcomes of patients with advanced infectious spondylitis treated with anterior corpectomy and reconstruction using a femoral ring allograft, followed by posterior instrumentation. We hypothesized that this technique would provide effective infection control and satisfactory structural stability without excessive complication rates.

## 2. Materials and methods

### 2.1. Study design and patients

This retrospective study was approved by the Institutional Review Board of Chonnam National University Hospital (CNUH-EXP-2024-336). The requirement for informed consent was waived owing to the retrospective design of the study. The study is reported in accordance with the Strengthening the Reporting of Observational Studies in Epidemiology statement for observational research.^[18]^ We reviewed the medical records of patients who underwent surgical treatment for thoracic or lumbar infectious spondylitis between December 2011 and September 2023. All procedures were performed by a single spine surgeon to reduce inter-operator variability. Patients were eligible for inclusion if they met the following criteria: advanced infectious spondylitis with significant vertebral body destruction requiring anterior corpectomy; anterior reconstruction using a femoral ring allograft followed by posterior instrumentation in a single-stage procedure; and availability of clinical and radiologic follow-up data for at least 12 months postoperatively.

Patients who underwent simple debridement without corpectomy, or anterior fusion without vertebral body resection, were excluded, as were those in whom the anterior column was reconstructed with a material other than a femoral ring allograft. Surgical site infection following instrumented spinal fusion was also excluded. Patients with <12 months of follow-up or with incomplete clinical or radiologic data were also excluded. Spondylitis occurring after percutaneous vertebral augmentation (vertebroplasty or kyphoplasty) was regarded as iatrogenic infectious spondylitis and was included in the analysis. The 2 situations were treated differently because they present different reconstructive problems. Infection following percutaneous vertebral augmentation leaves a destroyed vertebral body containing cement but no load-sharing posterior construct, so that corpectomy and anterior column reconstruction are required in exactly the same way as in other cases of advanced infectious spondylitis. By contrast, surgical site infection following instrumented spinal fusion arises in the presence of an established construct, in which decisions on implant retention, removal, or revision govern the surgical strategy and preclude an independent assessment of anterior column reconstruction. Of 139 patients screened, 114 were excluded, and 25 consecutive patients fulfilled the inclusion criteria and were included in the final analysis. The selection process is summarized in Figure 1.

**Figure 1.**
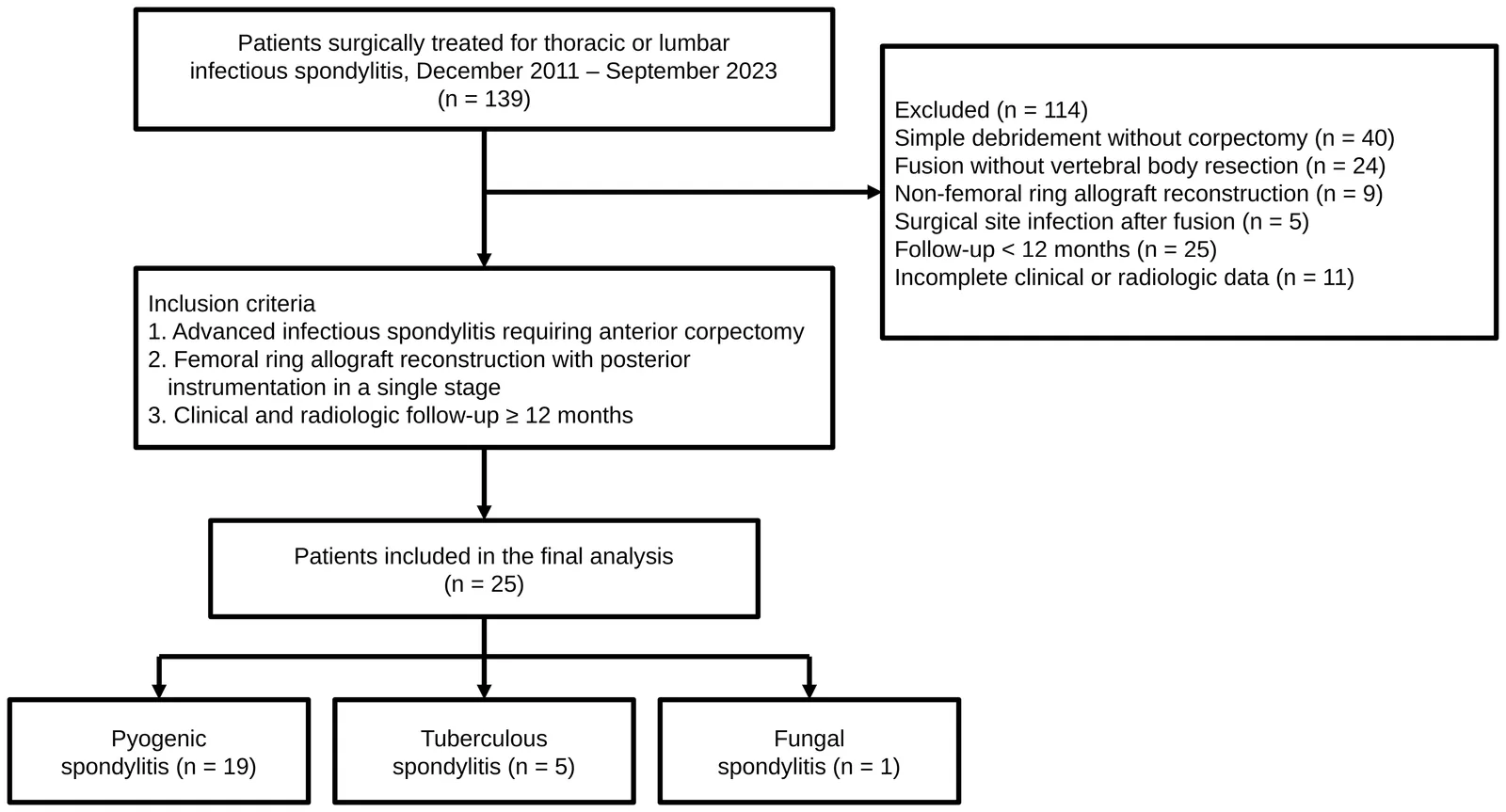
Flow diagram of patient selection. Of 139 patients who underwent surgical treatment for thoracic or lumbar infectious spondylitis between December 2011 and September 2023, 114 were excluded, and 25 were included in the final analysis. The analyzed patients are grouped by the type of infection for which they were treated: pyogenic (n = 19), tuberculous (n = 5), and fungal (n = 1). One culture-negative patient with concurrent pulmonary tuberculosis was managed as tuberculous spondylitis and is counted in the tuberculous subgroup; the same grouping is used for the treatment durations reported in Table 3 and the regimens in Table 1.

**Table 1:** Antimicrobial regimens according to culture results.

Pathogen	n	Parenteral therapy (wk)	Oral therapy (wk)	Total duration (wk)
Methicillin-resistant *Staphylococcus aureus*	2	Vancomycin (4)	Clindamycin (5–6)	9–10
Methicillin-sensitive *Staphylococcus aureus*	1	Cefazolin (4)	Cefroxadine (8)	12
Coagulase-negative *Staphylococcus*	3	Vancomycin or cefazolin (4–6)	Clindamycin or cefroxadine (4)	6–8
*Streptococcus* spp.	2	Cefazolin (4)	Cefroxadine (8–12)	12–16
Gram-negative bacilli (*Klebsiella pneumoniae*)	1	Cefotaxime (3)	Ciprofloxacin (5)	8
Anaerobe (*Parvimonas micra*)	1	Cefazolin (4)	Cefroxadine (16)	20
*Actinomyces odontolyticus*	1	Ceftriaxone (8)	–	8
Fungal (*Candida glabrata*)	1	Voriconazole (40)	–	40
*Mycobacterium tuberculosis*	4	–	Rifampicin and ethambutol ± isoniazid and pyrazinamide (40–50); second-line regimen with moxifloxacin, para-aminosalicylic acid, and prothionamide in 1 patient (38)	38–50
Culture-negative	9	Empirical cefazolin (2–4)	Cefroxadine or cefpodoxime proxetil (4–16)	4–40

Agents are given as international nonproprietary names and durations in weeks. All regimens were selected and supervised by the Division of Infectious Diseases and were individualized according to culture and susceptibility results, clinical response, and tolerability; no fixed institutional protocol was applied. Antituberculous therapy was administered orally throughout and therefore appears only in the oral column. The single patient with fungal spondylitis received parenteral voriconazole for the entire 40-week course without oral conversion. One culture-negative patient with concurrent pulmonary tuberculosis received a full oral antituberculous regimen for 40 weeks and is listed in the culture-negative row, which accounts for the upper limit of the total duration in that row. Among the 4 patients with *Mycobacterium tuberculosis*, 1 had concurrent pulmonary tuberculosis with spinal cultures positive for *M tuberculosis* and fully susceptible isolates on both molecular and phenotypic drug-susceptibility testing.

**Table 3:** Patient demographics and infection characteristics.

Parameter	n = 25
Sex (male/female)	8/17
Age (yr)	72.8 ± 11.5 (27–90)
Follow-up duration (mo)	29.2 ± 23.9 (12.0–87.2)
Iatrogenic (post-vertebral augmentation), n (%)	7 (28.0)
Pathogen, n (%)	
Culture-negative	9 (36.0)
* Mycobacterium tuberculosis*	4 (16.0)
Coagulase-negative *Staphylococcus*	3 (12.0)
Methicillin-resistant *Staphylococcus aureus*	2 (8.0)
* Streptococcus* spp.	2 (8.0)
Methicillin-sensitive *Staphylococcus aureus*	1 (4.0)
Gram-negative bacilli (*Klebsiella pneumoniae*)	1 (4.0)
Anaerobe (*Parvimonas micra*)	1 (4.0)
* Actinomyces odontolyticus*	1 (4.0)
Fungal (*Candida glabrata*)	1 (4.0)
Antimicrobial treatment duration (wk)	
Overall	18.3 ± 13.9 (4–50)
Pyogenic (n = 19)	11.0 ± 4.4 (4–20)
Tuberculous (n = 5)	41.6 ± 4.8 (38–50)
Fungal (n = 1)	40
Fusion level (segments)	3.3 ± 1.4 (2–7)
Ring filling material, n (%)	
Rib autograft ± DBM	14 (56.0)
Iliac autograft	10 (40.0)
DBM only	1 (4.0)
CT performed at final follow-up, n (%)	9 (36.0)

Values are presented as mean ± standard deviation (range) or number (%). CT = computed tomography, DBM = demineralized bone matrix. Antimicrobial treatment duration is presented separately by infection type because pyogenic and tuberculous regimens differ substantially in intended duration. One culture-negative patient with concurrent pulmonary tuberculosis received a full antituberculous regimen; this patient is counted within the tuberculous group for treatment duration and within the culture-negative group for pathogen distribution.

The diagnosis of infectious spondylitis was established based on persistent back pain, elevated inflammatory markers, and characteristic findings on magnetic resonance imaging, including vertebral body destruction, endplate erosion, disc space narrowing, and/or paravertebral or epidural abscess formation. Microbiological confirmation was obtained through blood cultures and/or intraoperative tissue cultures whenever possible. Surgical intervention was indicated in cases of progressive neurologic deficit, mechanical instability due to vertebral collapse, progressive deformity, abscess formation refractory to antibiotic therapy, or failure of conservative management.

All patients underwent anterior corpectomy and radical debridement via a transthoracic or retroperitoneal approach, depending on the lesion level. The choice of anterior column graft was determined intraoperatively by the size of the defect after radical debridement. A tricortical iliac autograft was used when the residual defect could be spanned by a single tricortical block, which in practice corresponded to defects confined to a single disc space with partial endplate loss. A femoral ring allograft was selected when the defect exceeded the dimensions that a tricortical iliac autograft could reliably support, typically after complete corpectomy of 1 or more vertebral bodies, or when the quantity of available autograft was judged insufficient for stable load-bearing reconstruction. Fresh-frozen femoral ring allografts were trimmed to fit the defect and used for structural anterior column support. The medullary cavity was filled primarily with locally harvested autologous bone (including morselized rib obtained during exposure) or demineralized bone matrix (DBM), and limited iliac cancellous bone was added selectively when necessary. Posterior stabilization was subsequently performed during the same operative session using open or percutaneous pedicle screw fixation. Postoperatively, all patients wore a thoracolumbosacral orthosis for 3 months.

All antimicrobial therapy was prescribed and supervised by the Division of Infectious Diseases. Empirical therapy was started after intraoperative specimens had been obtained and was subsequently tailored according to culture and antimicrobial susceptibility results, the clinical course, and serial inflammatory markers. Parenteral therapy was converted to oral therapy once clinical improvement and a sustained downward trend in C-reactive protein (CRP) had been confirmed, and treatment was continued until sustained normalization of inflammatory markers. Because regimens were individualized by the infectious disease specialists rather than following a fixed institutional protocol, both the agents used and the treatment duration varied among patients. Two patients had concurrent pulmonary tuberculosis; in one of these, spinal cultures were negative, but antituberculous therapy was administered on the basis of extraspinal microbiological confirmation. Antituberculous therapy was administered orally throughout, and the single patient with fungal spondylitis received parenteral voriconazole for the entire treatment period without oral conversion, so that the sequence of parenteral therapy followed by oral step-down applied only to the patients with pyogenic or culture-negative infection. The regimens actually used, grouped by culture result, are summarized in Table 1.

### 2.2. Assessments

Clinical outcomes were evaluated preoperatively and at the final follow-up (minimum 12 months postoperatively) using the Visual Analog Scale (VAS) for back pain, the Oswestry Disability Index (ODI), and the Frankel grading system to assess neurologic function.

Laboratory parameters, including erythrocyte sedimentation rate (ESR) and CRP, were measured preoperatively and serially during follow-up. For statistical analysis, preoperative and final follow-up values were compared.

Radiologic evaluation was performed using standing lateral and dynamic flexion–extension radiographs obtained preoperatively, immediately postoperatively, and at the final follow-up. Sagittal alignment was assessed by measuring the Cobb angle, defined as the angle formed between the superior endplate of the most cranial vertebra and the inferior endplate of the most caudal vertebra within the fused segment (Fig. 2). Changes between preoperative and final follow-up Cobb angles were analyzed to evaluate maintenance of sagittal correction. Fusion status was determined at the final follow-up based on dynamic radiographs. Solid fusion was defined as <2° of angular motion at the reconstructed segment, absence of radiolucent lines at the graft–endplate interface, and no evidence of implant loosening or graft collapse. Computed tomography (CT) was obtained at the final follow-up in 9 of the 25 patients (36.0%) at the discretion of the treating surgeon and was used to corroborate the radiographic assessment when available; CT was not performed routinely in all patients.

**Figure 2.**
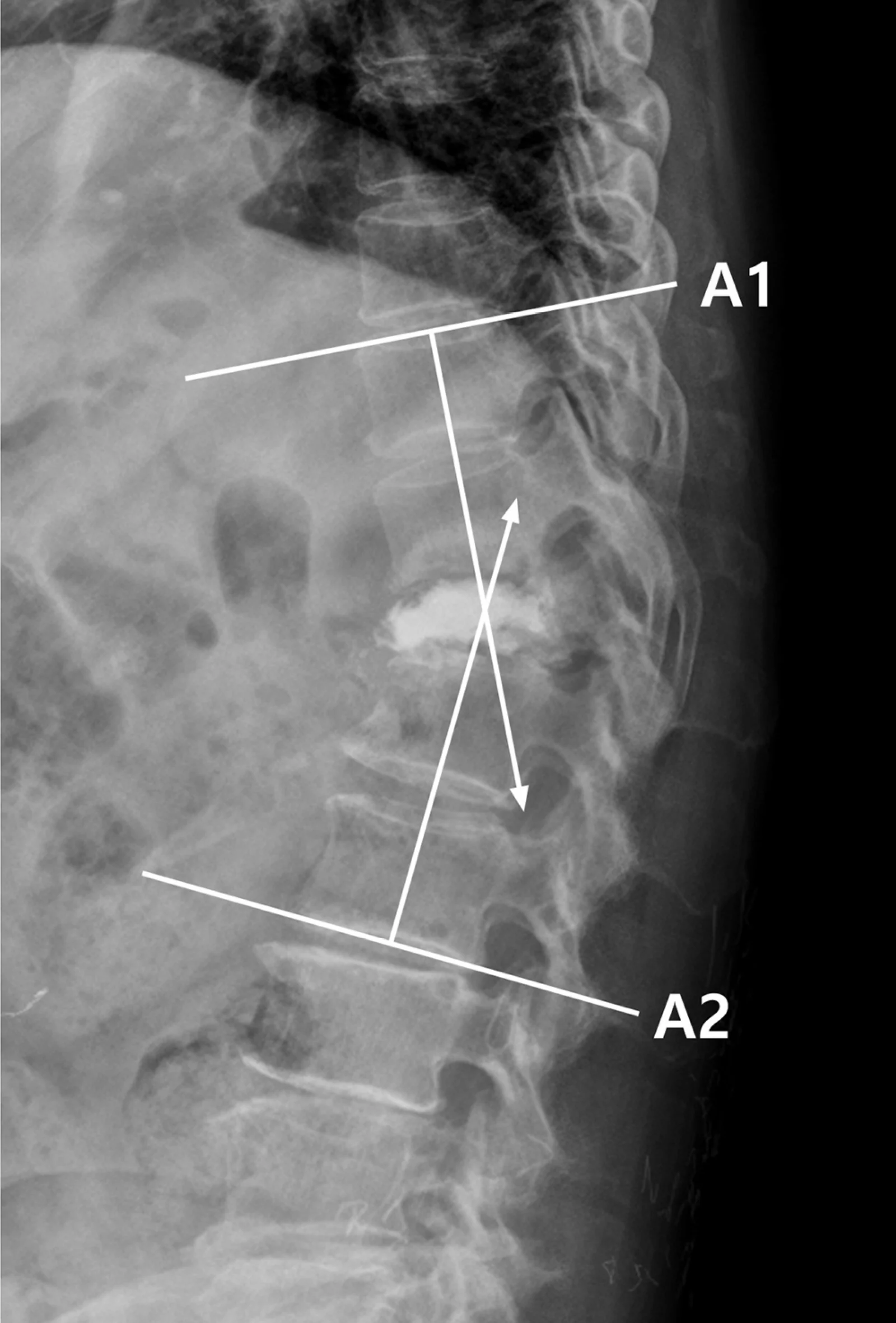
Measurement of sagittal alignment using the Cobb angle. The Cobb angle was defined as the angle between the superior endplate of the most cranial vertebra (A1) and the inferior endplate of the most caudal vertebra (A2) within the fused segment.

Infection control was defined as resolution of clinical symptoms, normalization or substantial decrease of inflammatory markers (CRP and/or ESR to within the normal reference range or to levels consistent with clinical recovery), absence of radiologic progression of infection, and no requirement for additional surgical intervention related to persistent or recurrent infection during follow-up. Complications were classified as infection-related, implant-related, or medical events and were recorded throughout the follow-up period. The timing, management, relationship to the surgical approach or reconstruction, and final outcome of each complication are presented in Table 2.

**Table 2:** Timing, management, and outcome of the 4 complications.

Case	Category	Event	Onset	Management	Relationship to approach or reconstruction	Outcome at final follow-up
1	Implant-related	Fixation failure	3 mo	Implant removal	Related to the limited number of levels in the construct (2-level fixation at the thoracolumbar junction), not to the percutaneous technique	Resolved; no further kyphotic progression until final follow-up
2	Infection-related	Recurrent infection	60 mo	Nonoperative; further surgery declined by the family because of advanced age	Related to fungal (*Candida glabrata*) infection in an elderly patient; not related to the graft material or the surgical approach	Infection not controlled; subsequently lost to follow-up
3	Medical	Postoperative pneumothorax	3 d	Oxygen supplementation and observation	Related to the transthoracic approach	Asymptomatic; resolved without sequelae
4	Medical	Antibiotic-induced cytopenia	2 wk	Change of antimicrobial agent to daptomycin	Related to prolonged antimicrobial exposure, not to the procedure	Asymptomatic; resolved

Onset is expressed as the interval between the index operation and the first documented occurrence of the event.

### 2.3. Statistical analysis

Continuous variables are presented as mean and standard deviations, while categorical variables are expressed as percentages. Preoperative and postoperative values for VAS, ODI, ESR, CRP, and Cobb angle were compared using the Wilcoxon signed-rank test given the small sample size and non-normal distribution. Frankel grades were treated as paired ordinal variables and analyzed using the same method. Because significance testing alone conveys neither the magnitude nor the precision of the observed changes in a small cohort, the Hodges–Lehmann estimate (the median of all pairwise differences between the final follow-up and preoperative values) was calculated for each paired comparison together with its distribution-free 95% confidence interval (CI), and the effect size *r* was computed as |*Z*|/√N, where values of 0.1, 0.3, and 0.5 correspond to small, medium, and large effects, respectively. All statistical analyses were performed using SPSS software version 18.0 (SPSS Inc., Chicago), with Hodges–Lehmann estimates and their CIs obtained in R version 4.3.1 (R Foundation for Statistical Computing, Vienna, Austria). A *P*-value < .05 was considered statistically significant.

## 3. Results

A total of 25 patients were included in the final analysis of this study. There were 8 men and 17 women, with a mean age of 72.8 ± 11.5 years (range, 27–90 years). The mean follow-up duration was 29.2 ± 23.9 months (range, 12.0–87.2 months), with a median of 24.0 months; 4 patients were followed for more than 5 years. The mean duration of antimicrobial therapy was 18.3 ± 13.9 weeks (range, 4–50 weeks) overall and differed substantially by infection type: 11.0 ± 4.4 weeks (range, 4–20 weeks) in pyogenic infection, 41.6 ± 4.8 weeks (range, 38–50 weeks) in tuberculous infection, and 40 weeks in the single fungal infection (Table 3).

The causative pathogen was identified in 16 patients (64.0%), while 9 patients (36.0%) had culture-negative infections. Among the identified pathogens, *Mycobacterium tuberculosis* was the most common (n = 4, 16.0%), followed by coagulase-negative *Staphylococcus* (n = 3, 12.0%), methicillin-resistant *Staphylococcus aureus* (n = 2, 8.0%), and *Streptococcus* spp. (n = 2, 8.0%). Methicillin-sensitive *S aureus*, Gram-negative bacilli (*Klebsiella pneumoniae*), an anaerobe (*Parvimonas micra*), *Actinomyces odontolyticus*, and a fungal pathogen (*Candida glabrata*) were each identified in 1 patient (4.0%; Table 3). Seven patients (28.0%) had iatrogenic spondylitis that developed after percutaneous vertebral augmentation.

The affected spinal levels ranged from T10 to L5. The mean number of fused segments was 3.3 ± 1.4 (range, 2–7). Regarding graft filling material, rib autograft with or without DBM was used in 14 patients (56.0%), iliac autograft in 10 patients (40.0%), and DBM alone in 1 patient (4.0%; Table 3). CT was obtained at the final follow-up in 9 patients (36.0%).

Significant improvements in clinical outcomes were observed at the final follow-up. Clinical, laboratory, and radiologic outcomes are summarized in Table 4. The mean VAS score for back pain improved from 7.3 ± 1.1 preoperatively to 1.9 ± 1.0 at the final follow-up (Hodges–Lehmann estimate −5.50; 95% CI, −6.00 to −5.00; *r*  = 0.87; *P* < .001). The mean ODI also decreased from 37.7 ± 5.9 to 10.7 ± 3.5 (−26.50; 95% CI, −29.50 to −23.50; *r* = 0.87; *P* < .001).

**Table 4:** Clinical, laboratory, and radiologic outcomes.

Parameter	Preoperative	Final follow-up	Hodges–Lehmann estimate (95% CI)	Effect size *r*	*P* value
VAS (back pain)	7.3 ± 1.1	1.9 ± 1.0	−5.50 (−6.00 to − 5.00)	0.87	<.001
ODI (%)	37.7 ± 5.9	10.7 ± 3.5	−26.50 (−29.50 to − 23.50)	0.87	<.001
CRP (mg/dL)	6.43 ± 7.49	0.29 ± 0.41	−4.75 (−7.01 to − 2.77)	0.86	<.001
ESR (mm/h)	70.4 ± 25.7	34.9 ± 19.4	−36.00 (−49.50 to − 23.00)	0.79	<.001
Cobb angle (°)	−10.9 ± 14.2	−4.7 ± 11.9	5.55 (3.40–8.95)	0.69	<.001
Frankel grade (A/B/C/D/E)	0/1/10/13/1	0/0/1/10/14	1.00 (0.50–1.00)	0.76	<.001
Fusion achievement, n (%)	–	23 (92.0)	–	–	–
Infection control, n (%)	–	24 (96.0)	–	–	–
Complications, n (%)	–	4 (16.0)	–	–	–

Values are presented as mean ± standard deviation unless otherwise indicated. All paired comparisons were performed using the Wilcoxon signed-rank test. The Hodges–Lehmann estimate is the median of all pairwise differences (final follow-up minus preoperative) with its distribution-free 95% confidence interval. Effect size *r* was calculated as *Z* /√N (N = 25); values of 0.1, 0.3, and 0.5 correspond to small, medium, and large effects, respectively. Frankel grade was analysed as a paired ordinal variable, and its Hodges–Lehmann estimate is expressed in grade increments.

CI = confidence interval, CRP = C-reactive protein, ESR = erythrocyte sedimentation rate, ODI = Oswestry Disability Index, VAS = visual analogue scale.

Neurological function also improved. Preoperatively, 1 patient was classified as Frankel grade B, 10 as grade C, 13 as grade D, and 1 as grade E. At the final follow-up, 1 patient remained in grade C, 10 were classified as grade D, and 14 as grade E (Hodges–Lehmann estimate 1.00 grade; 95% CI, 0.50–1.00; *r* = 0.76; *P* < .001). Nineteen of the 25 patients (76.0%) improved by at least 1 Frankel grade, and no patient deteriorated neurologically.

Laboratory parameters demonstrated significant improvement. The mean ESR decreased from 70.4 ± 25.7 mm/h preoperatively to 34.9 ± 19.4 mm/h at the final follow-up (−36.00; 95% CI, −49.50 to −23.00; *r* = 0.79; *P* < .001), while the mean CRP level decreased from 6.43 ± 7.49 mg/dL to 0.29 ± 0.41 mg/dL (−4.75; 95% CI, −7.01 to −2.77; *r* = 0.86; *P* < .001).

Radiologic assessment demonstrated maintenance of sagittal alignment following surgery. The mean Cobb angle improved from −10.9  ± 14.2° preoperatively to 1.4 ± 12.8° immediately after surgery, corresponding to an initial correction of approximately 12.2°. During follow-up, a partial loss of correction was observed, with the Cobb angle measuring −4.7 ± 11.9° at the final follow-up, representing a correction loss of approximately 6.1°. Nevertheless, the final alignment remained improved compared with the preoperative state, with a net correction of approximately 6.1° (Hodges–Lehmann estimate 5.55°; 95% CI, 3.40°–8.95°; *r* = 0.69; *P* < .001). A representative case demonstrating initial correction and subsequent partial loss of alignment is shown in Figure 3.

**Figure 3.**
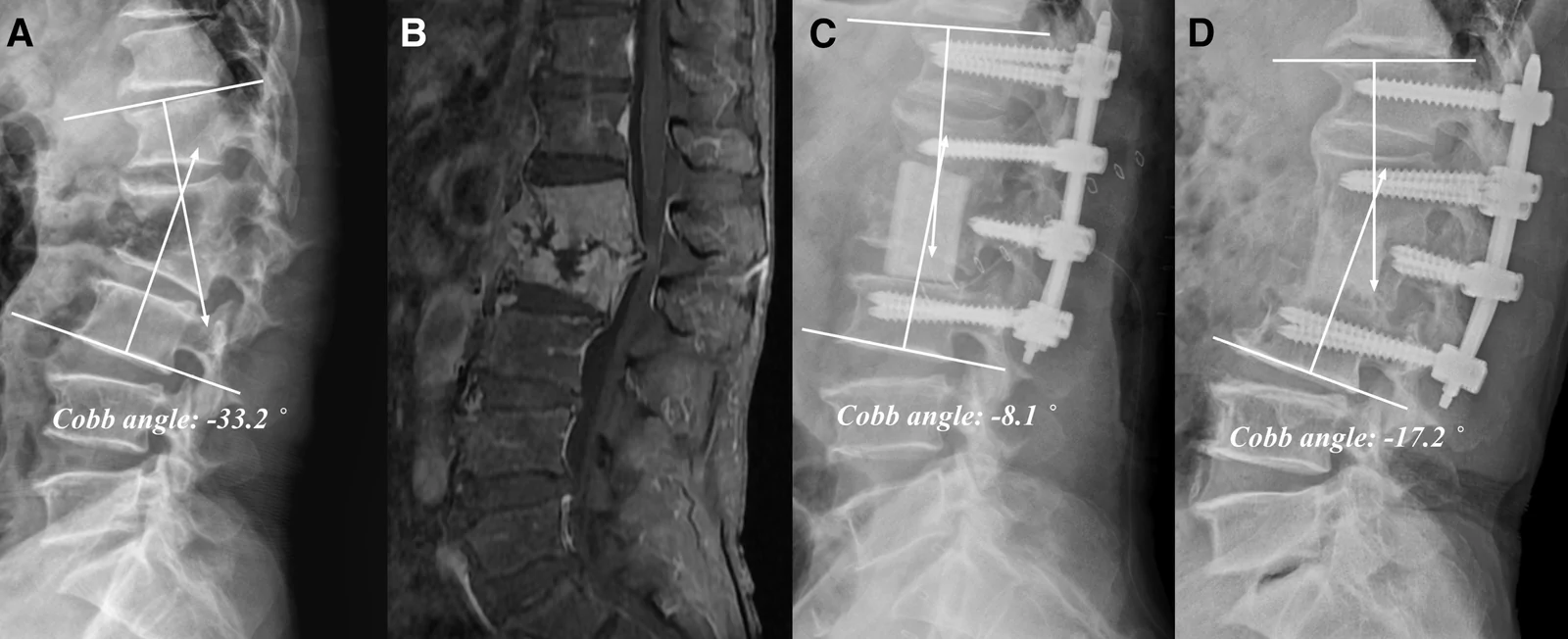
Representative case demonstrating sagittal alignment correction and subsequent partial loss. (A) Preoperative lateral radiograph showing marked kyphotic deformity. (B) Preoperative gadolinium-enhanced magnetic resonance imaging demonstrating infectious spondylitis with vertebral body destruction. (C) Immediate postoperative lateral radiograph showing restoration of sagittal alignment after anterior reconstruction and posterior instrumentation. (D) Final follow-up lateral radiograph (80.0 months) demonstrating partial loss of correction with solid fusion, with overall alignment remaining improved compared with the preoperative state.

Solid fusion was achieved in 23 of 25 patients (92.0%) at the final follow-up. Infection control was achieved in 24 of 25 patients (96.0%). One patient (4.0%) developed recurrent infection 60 months after surgery, presenting with a draining fistula over the posterior wound; further surgical treatment was declined by the family because of the patient’s advanced age, and the infection remained uncontrolled at the time the patient was lost to follow-up. Overall complications occurred in 4 patients (16.0%). These included 1 infection-related complication (the recurrent infection described above), 1 implant-related complication (fixation failure), and 2 medical complications, namely postoperative pneumothorax on the third postoperative day and antibiotic-induced cytopenia at 2 weeks. The timing, management, relationship to the surgical approach or reconstruction, and final outcome of each event are detailed in Table 2.

## 4. Discussion

The present study demonstrated that anterior reconstruction using femoral ring allografts following corpectomy provides effective infection control, satisfactory fusion, and acceptable complication rates in patients with advanced infectious spondylitis. Although femoral ring allografts have not been widely used in infected spinal conditions, our findings suggest that they can be a viable alternative for anterior column reconstruction in this setting. To our knowledge, this is one of the largest clinical series evaluating femoral ring allograft reconstruction in infectious spondylitis, and the mean follow-up of 29.2 months, with 4 patients followed beyond 5 years, provides some information on the medium-term behavior of these grafts.

The first-line treatment for infectious spondylitis is conservative management with antimicrobial therapy.^[7]^ However, surgical intervention becomes necessary in cases where medical treatment fails, neurological deficits are present, or other specific indications exist.^[2]^ In cases of extensive infection and significant bony destruction, anterior corpectomy combined with posterior instrumentation is often required.^[7]^ A recent Korean series comparing access routes found that an anterior approach, with or without posterior instrumentation, was associated with fewer treatment failures and fewer unplanned revision procedures than a posterior-only approach, which supports the reconstruction strategy used here.^[19]^

In infectious spondylitis, reconstruction options following corpectomy include autografts, metal cages, and allografts.^[8]^ Autografts are generally considered the gold standard due to their osteogenic potential and resistance to infection. However, harvesting structural autografts is associated with donor-site morbidity and limited graft availability, particularly in elderly patients or in cases requiring long-segment reconstruction.^[9]^ Therefore, alternative reconstructive options have been explored.

Although the use of implants in an infected field has traditionally been considered contraindicated, several studies have demonstrated the safe and effective use of metal cages and allografts in the treatment of infectious spondylitis.^[11,20-22]^ Structural allografts, including fibular or tibial strut grafts, have shown favorable outcomes in terms of fusion and infection control.^[12-14]^ Contemporary practice has shifted substantially toward titanium-based reconstruction. Two recent meta-analyses of spinal tuberculosis found no significant difference between titanium mesh grafting and structural bone grafting in fusion, deformity correction, neurological recovery, or complication rates, although the rate of implant subsidence was marginally lower with bone grafting.^[15,16]^ Three-dimensional printed porous titanium cages have more recently been introduced, but a 2026 systematic review concluded that the direct evidence in infectious spondylodiscitis remains limited and heterogeneous and does not yet demonstrate superiority over conventional interbody devices.^[23]^ Structural allograft therefore remains a defensible option in this setting, particularly where cost, implant availability, or the preference to avoid a permanent metallic implant in an infected field are relevant considerations. In the largest contemporary series of structural bone allograft in spinal surgery, comprising 147 patients followed for a mean of more than 12 years, fusion was achieved and maintained in 98.9% of cervical and 97.9% of lumbar reconstructions with a single case of subsidence, indicating that allograft incorporation, once achieved, is durable well beyond the observation period of most spinal infection series.^[24]^

Several studies have reported the use of femoral allografts in spinal surgery.^[17,25,26]^ More recently, Chang et al reported that structural femoral shaft allografts used for anterior column reconstruction in osteoporotic spines achieved significant kyphosis correction with minimal graft subsidence, attributing this to the morphometric and biomechanical advantages of cortical femoral bone over metallic implants at weakened endplates – a consideration of particular relevance to the elderly cohort treated here.^[27]^ However, their application in infectious spondylitis remains limited. Compared with commonly used structural grafts such as fibular or tibial strut allografts,^[12,13]^ femoral ring allografts have been less frequently reported in this setting, possibly due to historical preferences and technical considerations. A recent series in which circumferential debridement and anterior reconstruction with a fibular allograft were performed through a single posterior approach achieved eradication of infection in all patients without fixation failure, with improvements in VAS and ODI scores comparable to those observed here, indicating that structural allograft remains an actively used reconstruction option.^[28]^ In the present study, we demonstrated favorable clinical and radiologic outcomes using femoral ring allograft struts, supporting their applicability in the surgical management of complex infectious spondylitis.

In this study, patients presented with advanced infectious spondylitis, including cases with vertebral body collapse and infection following vertebroplasty or kyphoplasty, which accounted for 7 of the 25 patients (28.0%) and reflects the increasing use of percutaneous vertebral augmentation in elderly patients with osteoporotic fractures, requiring corpectomy and reconstruction. The femoral ring allografts were primarily filled with autologous bone, including rib or iliac cancellous grafts, except for 1 case in which only DBM was used. This approach is similar to the hybrid graft technique described by El-Masry et al, which has demonstrated favorable outcomes.^[26]^

Postoperative improvements in pain and functional outcomes, as reflected by VAS and ODI scores, were significant, and the corresponding effect sizes were large (*r* = 0.87 for both), indicating that the magnitude of improvement, and not only its statistical significance, was substantial. Neurological function also improved in most patients, as demonstrated by improvements in Frankel grades in 19 of 25 patients (76.0%), with no neurological deterioration. These findings are in line with previous reports emphasizing the role of adequate decompression and stabilization in achieving favorable outcomes in infectious spinal pathology.^[5,12]^

The significant reductions in CRP and ESR levels observed in this study indicate effective infection control.^[29]^ These findings support the concept that thorough surgical debridement combined with targeted antimicrobial therapy plays a crucial role in the successful management of spinal infections. In our cohort, parenteral therapy in pyogenic infection was continued for a median of 4 weeks (range, 2–4 weeks) before oral step-down in the 16 patients in whom the parenteral and oral phases were separately recorded, which is shorter than the 6 weeks of parenteral therapy recommended by the Infectious Diseases Society of America; conversion was, however, undertaken only after radical debridement and reconstruction and after a sustained fall in CRP, and the total duration of treatment averaged 11.0 weeks.^[30]^

Radiologic evaluation demonstrated that substantial correction of sagittal alignment was achieved immediately after surgery. However, a partial loss of correction was observed during follow-up. This finding likely reflects graft subsidence and remodeling in the setting of weakened vertebral endplates due to infection. Nevertheless, the final alignment remained improved compared with the preoperative state. This pattern of initial correction followed by partial loss, likely related to graft subsidence and remodeling, has been reported in studies involving structural graft reconstruction in infectious spondylitis and is generally considered clinically acceptable.^[12,13,27]^ In the present cohort, the mean initial correction of 12.2° was followed by a loss of 6.1°, leaving a net correction of 6.1°.

Despite the overall favorable outcomes, complications were observed in 4 patients, the details of which are presented in Table 2. One case of fixation failure occurred early in the surgical experience and involved only 2-level percutaneous fixation at the thoracolumbar junction, which may have provided insufficient stability (Fig. 4); the failure was identified on the 3-month follow-up radiograph, the construct was removed, and no further kyphotic progression was observed until the final follow-up. Recent evidence indicates that percutaneous and open pedicle screw fixation yield comparable outcomes in pyogenic spondylodiscitis, which suggests that the number of levels incorporated into the construct, rather than the percutaneous technique itself, was the relevant factor; a longer construct extending over additional levels above and below the reconstruction would probably have been more appropriate for a lesion of this extent at the thoracolumbar junction.^[31]^ The single case of infection control failure occurred in an 81-year-old patient with *C glabrata* spondylitis who developed a draining fistula 60 months after surgery; further surgical treatment was declined by the family because of the patient’s age and comorbidity, so no revision procedure was performed, and the infection remained uncontrolled at the time of loss to follow-up. Fungal spondylodiscitis is uncommon, is associated with a higher rate of relapse than pyogenic infection, and typically requires prolonged antifungal therapy, which may partly explain this outcome. For context, a Korean nationwide cohort of 2148 patients who underwent early instrumented fusion for pyogenic spondylodiscitis reported a recurrence rate of 10%, with age over 60 years among the factors most strongly associated with recurrence; the 4% recurrence rate observed here is therefore within, and at the lower end of, the reported range for a cohort of this age profile.^[32]^ In addition, 1 patient developed antibiotic-induced cytopenia during antimicrobial therapy, which was likely related to prolonged antibiotic exposure and resolved after adjustment of the medication regimen to daptomycin at 2 weeks. One patient experienced postoperative pneumothorax on the third postoperative day, which was considered to be associated with the surgical approach (transthoracic exposure) and was managed with supplemental oxygen and observation without long-term sequelae.

**Figure 4.**
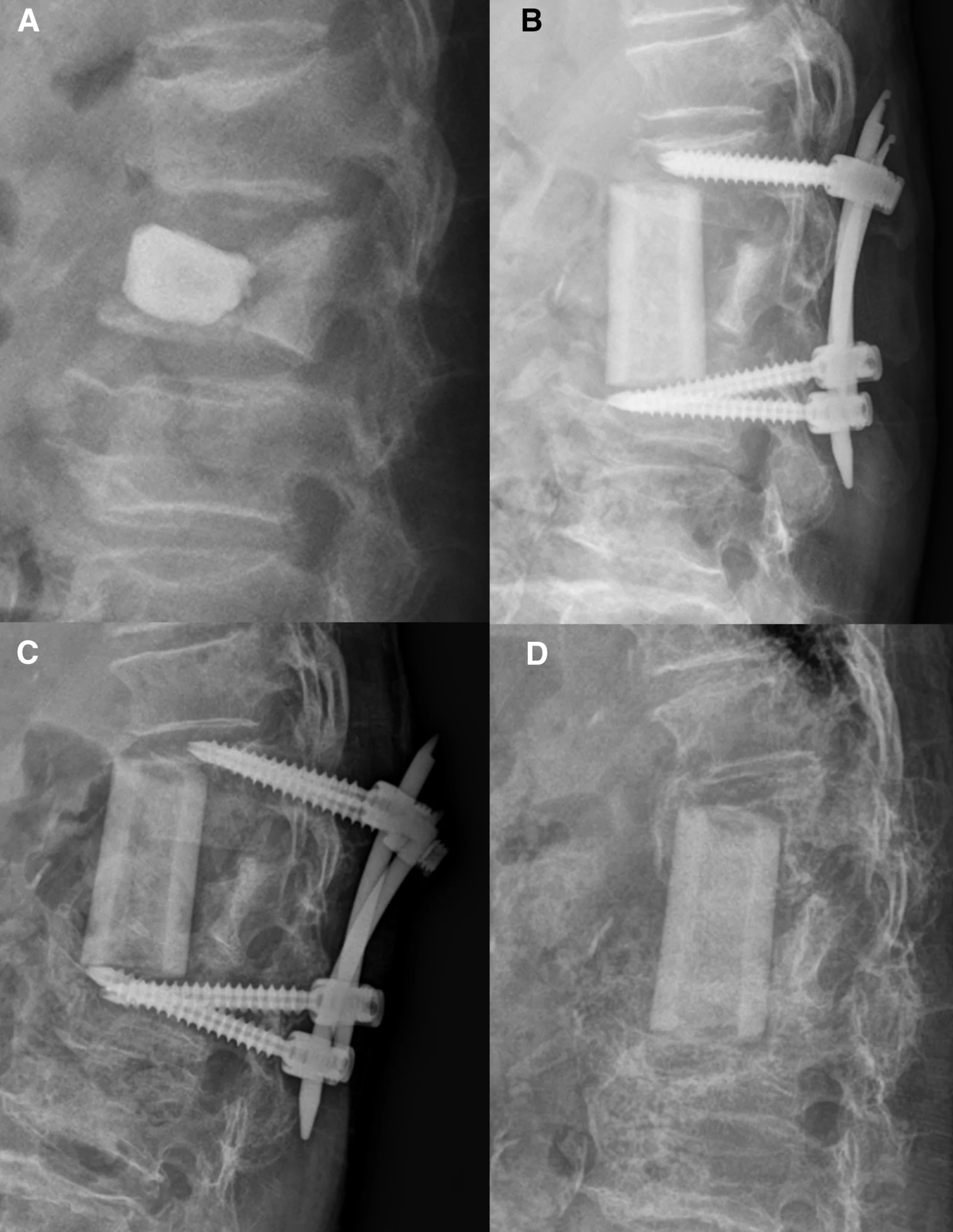
Representative case of fixation failure. (A) Preoperative lateral radiograph showing vertebral body destruction following previous kyphoplasty. (B) Immediate postoperative radiograph demonstrating anterior reconstruction with femoral ring allograft and 2-level posterior fixation. (C) Three-month follow-up radiograph showing fixation failure with loss of alignment. (D) Radiograph after implant removal demonstrating pain relief without additional instrumentation.

In our cohort, culture-negative infections accounted for 36.0% of cases. This proportion is higher than in some surgical series but lies within the 25% to 50% range reported for culture-negative native vertebral osteomyelitis, in which prior antimicrobial exposure is the most consistently identified contributing factor.^[33-35]^ Culture-negative disease has been reported to follow a more indolent course than microbiologically confirmed infection, which underlines the importance of obtaining adequate tissue for culture before antimicrobial therapy is started. This finding is consistent with our prior study by Sung et al, which suggested that low microbiological yield in spinal infections is frequently associated with prior antibiotic exposure at referring institutions, leading to reduced bacterial recovery.^[36]^ In the same study, the clinical presentation of infectious spondylitis was also noted to be often nonspecific and may overlap with degenerative spinal conditions. Therefore, in such cases, diagnostic and therapeutic decisions should be based on an integrated assessment of clinical presentation, inflammatory markers, and characteristic imaging findings rather than microbiological confirmation alone.

This study has several limitations. First, this was a retrospective study of 25 patients without a control group. A comparison with the tricortical iliac autografts used at our institution over the same period would not have been informative because the choice of graft was determined by the size of the defect remaining after debridement rather than by allocation: femoral ring allografts were reserved for defects that a tricortical autograft could not span, and the patients reconstructed with a tricortical autograft did not undergo corpectomy at all. Any comparison between the 2 groups would therefore reflect the extent of vertebral destruction rather than the reconstructive material, a form of confounding by indication that cannot be removed by adjustment in a cohort of this size. Anterior reconstruction with a metal cage was performed in only a few patients with comparable defects, too few for meaningful comparison. Second, there were variations in surgical techniques and causative pathogens among the study subjects. While all cases involved single-stage anterior corpectomy and reconstruction for complicated infectious spondylitis, the extent of posterior instrumentation varied. In addition, the materials used to fill the femoral ring struts included rib autografts, iliac cancellous autografts, and DBM. The causative pathogens also varied, including bacteria, tuberculosis, and fungi, with some cases requiring empirical antibiotic treatment due to unidentified pathogens. Third, antimicrobial regimens were individualized by the infectious disease service rather than following a fixed protocol, so both the agents and the treatment durations were heterogeneous, and the effect of this variability on outcome cannot be separated from that of the surgical treatment. Fourth, although the mean follow-up of 29.2 months and the inclusion of 4 patients followed beyond 5 years provide more information than a strictly short-term series, the follow-up was still of limited duration and unevenly distributed, with a median of 24.0 months. Late graft resorption, delayed subsidence, and infection recurrence beyond the observation period may therefore have been underestimated; notably, the single recurrence in this cohort occurred at 60 months, which would have been missed had follow-up been limited to 2 years. Series reporting more than a decade of follow-up after structural allograft reconstruction suggest that incorporation, once achieved, is maintained,^[24]^ but this cannot be confirmed from the present data. Finally, radiologic fusion was primarily assessed using dynamic radiographs, and CT was not consistently performed in all patients, having been obtained at final follow-up in only 9 of 25 patients (36.0%), which may have led to overestimation of the fusion rate.

## 5. Conclusion

In patients with advanced infectious spondylitis and large anterior column defects that could not be reconstructed with a tricortical iliac autograft, anterior reconstruction using a femoral ring allograft following corpectomy achieved reliable infection control, a high fusion rate, and satisfactory clinical and radiologic outcomes over a mean follow-up of 29.2 months, with an acceptable complication profile. Within this follow-up period and for this specific size-based indication, the technique appears to be a reasonable alternative to structural autograft when donor-site morbidity or graft availability is a concern. Longer-term and directly comparative studies are required to confirm the durability of graft incorporation, to characterize late subsidence and recurrence, and to define the role of this technique relative to contemporary titanium-based reconstruction.

## Acknowledgments

This research was supported by Chonnam National University (Grant number 2025-0367-03).

## Author contributions

**Conceptualization:** Hyoung-Yeon Seo.

**Data curation:** Myung-Jin Sung.

**Formal analysis:** Chun-Gon Park.

**Investigation:** Myung-Jin Sung.

**Methodology:** Chun-Gon Park.

**Resources:** Myung-Jin Sung.

**Supervision:** Hyoung-Yeon Seo.

**Validation:** Chun-Gon Park.

**Visualization:** Chun-Gon Park.

**Writing – original draft:** Myung-Jin Sung.

**Writing – review & editing:** Myung-Jin Sung, Hyoung-Yeon Seo.
